# A Rare Case of Gorham’s Disease: Primary Ulnar Involvement with Secondary Spread to the Radius and Elbow

**DOI:** 10.5812/traumamon.9905

**Published:** 2013-05-26

**Authors:** Reza Tavakoli Darestani, Amir Sharifzadeh, Mohammadmahdi Bagherian Lemraski, Ramin Farhang Zanganeh

**Affiliations:** 1 Department of Orthopedics, Imam Hossein Medical Center, Shahid Beheshti University of Medical Sciences, Tehran, IR Iran

**Keywords:** Osteolysis,Essential, Ulna, Forearm

## Abstract

**Introduction:**

Gorham’s disease is a rare musculoskeletal disease which causes progressive osteolysis and is characterized by massive bone destruction due to proliferation of vascular elements along with a great number of osteoclasts. The etiology of the disease is unknown. Gorham’s disease is essentially rare in the forearm bones. As far as we know, only 2 cases of Gorham’s disease of the forearm have been reported with 1 of them in the radius and the other starting in the radius and spreading to the lower portion of the humerus.

**Case Presentation:**

This case report shows that Gorham’s disease may affect the ulna primarily and spread to adjacent bones despite the fact that there are no such reports in the literature.

**Conclusions:**

Gorham’s disease has several manifestations as primary bone involvement. As in this rare case the ulna may be affected first and then the disease may spread to adjacent bones. More studies are needed to better recognize the behavior of this rare disease.

## 1. Introduction

There are many diseases that cause significant osteolysis. These syndromes differ according to the mode of transmission, accompanying signs and symptoms and the location of osteolysis (1). Gorham’s disease is a rare musculoskeletal disease which causes progressive osteolysis (1-3). This disease was first reported in 1838 by Jackson (4-7). Later in 1954 Gorham et al. reported 2 cases of the disease. In 1955 Gorham and Stout described the clinical and pathological manifestations of the disease as an osteolytic process and the disease was named “Gorham’s disease” in the honor of LW Gorham (4-7). Gorham’s disease is characterized by massive bone destruction due to proliferation of vascular elements along with a great number of osteoclasts (2-8). Angiomatosis and sometimes lymphatic proliferation, soft tissue swelling and lack of osteogenesis are the main features of the disease (1-9). The etiology and pathogenesis of the disease are unknown (10). Other names of the disease include massive osteolysis disease, phantom bone disease and vanishing bone disease (9-11). Gorham’s disease may affect any bone in the body but usually bones that are formed by intramembranous ossifications such as the pelvic and the shoulder girdle are affected (7, 8, 12). As many as 200 cases have been reported up to now affecting the skull, maxillofacial bones, pelvis, ribs, sternum, femur, foot, hand, humerus, spine, scapula and the clavicle (1-10). As far as we know, only 2 cases of Gorham’s disease in the forearm have been reported (10-13) with 1 of them in the radius (13) and the other starting in the radius and spreading to the lower portion of the humerus (10).

## 2. Case Presentation

**Figure 1.**
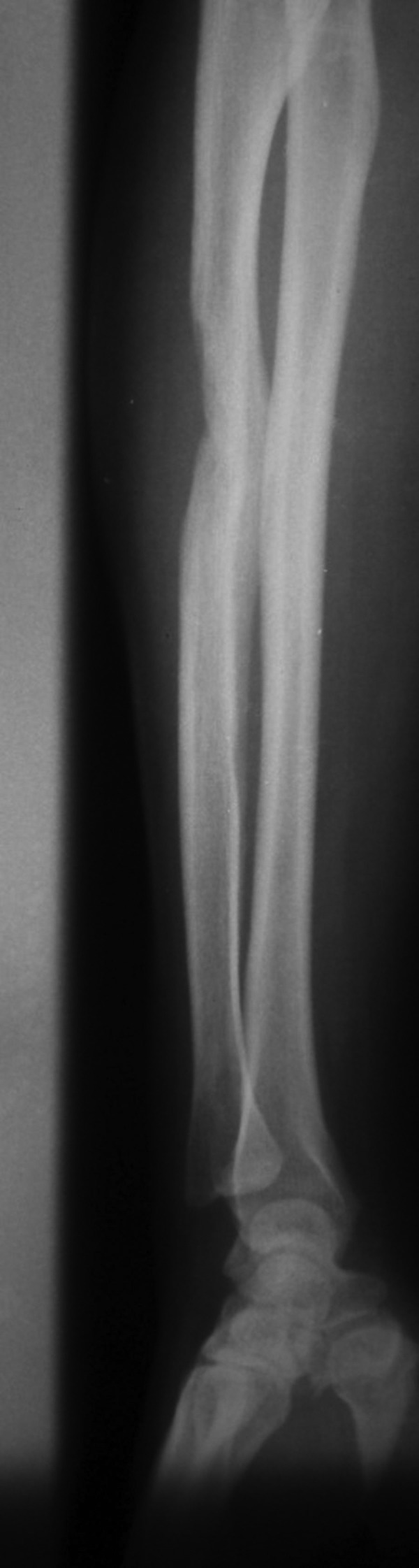
Anterior Posterior View of the Forearm Showing Resorption of Part of the Ulna

**Figure 2.**
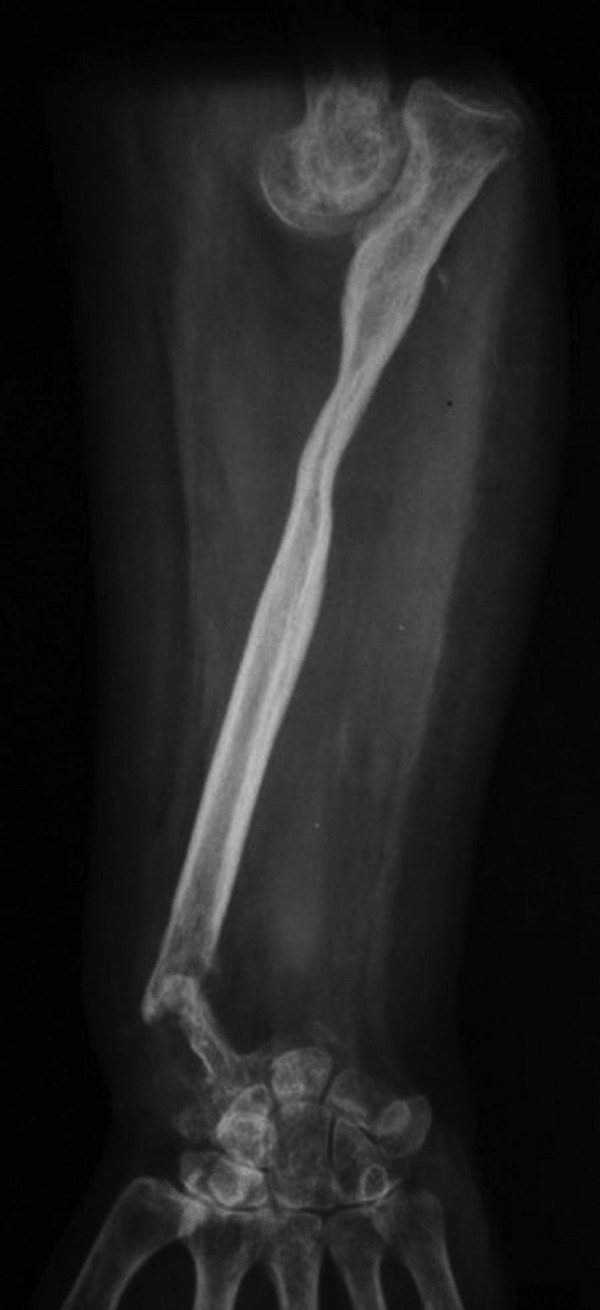
Anterior, Posterior and Lateral Views of the Forearm Showing Fracture of the Ulna

**Figure 3.**
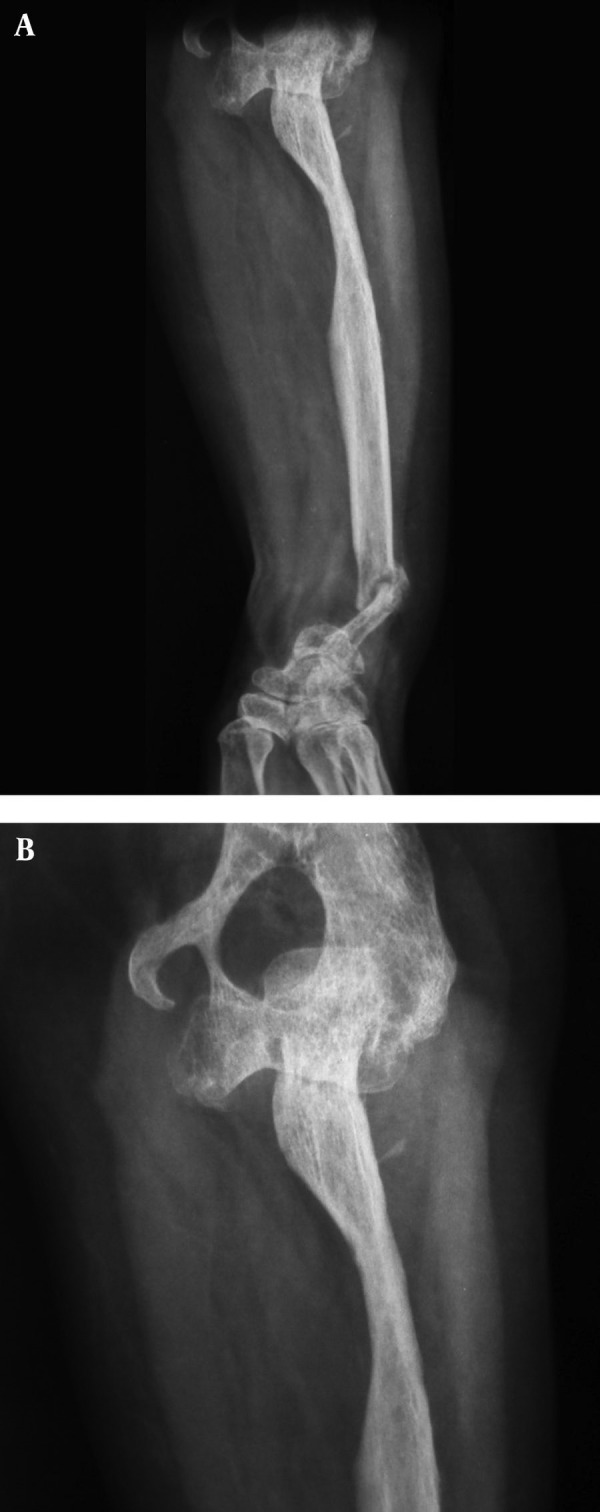
These Figures Show Resorption of the Entire Ulna Also Fracture and Resorption of Distal Part of the Radius. A) Anterior posterior view of forearm in pronation; B) Lateral view of forearm in pronation

A 60 y/o female presented with Gorham’s disease in the ulna. The disease first started in the ulna and later involved the lower end of the radius and the lower humerus. The patient had referred to her doctor 9 years earlier with pain in the ulnar side of her forearm after getting hit by a heavy object. Clinical and radiographic examinations had failed to reveal any particular pathology and the patient was then referred to a physiotherapist. One year later, the patient returned to her physician with severe pain in the forearm. Plain X-rays were apparently normal, however MR imaging revealed nonspecific lesions in the ulnar bone marrow. These lesions were suspected to be chronic osteomyelitis. Radionuclide and CT scans revealed low density areas in the ulna. Laboratory, microbiological, immunological, hormonal and biochemical tests, including parathyroid tests were all within normal limits. The patient had a biopsy taken by her orthopedic surgeon. The biopsy was apparently consistent with the diagnosis of osteomyelitis, but the culture returned negative. After a while, the patient sustained a fracture in the same bone while asleep which was treated with a cast and healed without surgery. The patient has been complaining of pain and weakness in the left forearm for the past 5 years. Radiographs revealed bone absorption in parts of the ulna (Figure 1). Five months ago, while attempting to lift a glass, the patient twisted her forearm and broke her radius. Her radiographs showed complete resorption of the ulna (Figure 2); her arm was subsequently immobilized in a long arm cast. She came to our center and radiographs of her arm revealed non-union of her latest fracture. They also showed bony absorption of the lower end of the radius (Figures 3 A,B and C). The patient's elbow ROM was nearly normal, so was the wrist ROM except for the final degression of flexion and extension. MTPs and finger motion were normal and the patient's fingers were spared.The neurovascular status had been normal from the onset of the disease and the nerves and vessels were spared. The skin was normal and no soft tissue involvement was seen. All laboratory tests including serum complement levels, vasculitis tests, parathyroid, 24-hour urinary proteins and electrophoresis of plasma proteins were normal. Based on test results and the histopathology report of dead, edematous bony tissue and an abundance of inflammatory cells with a great number of thick-walled blood vessels and no evidence of malignancy, we considered the criteria proposed by Heffez et al. in 1983 ( 14 ) and Gorham’s disease was diagnosed.

## 3. Conclusions

Gorham’s disease is an extremely rare disease that has remained unexplained despite many years after its first description by Gorham and Stout. This disease has been reported in various locations previously, but as far as we have searched, our case is the first case of Gorham’s disease that has primarily affected the ulna. It should be mentioned that this disease is essentially rare in the forearm bones. Involvement of the radius has been reported in a number of occasions namely in 1997 Spieth et al. reported a case of Gorham’s disease in the forearm of a 46 year-old woman (13). In 2008 Rubel et al. reported primary Gorham’s disease in the radius of a 47 year-old woman that later involved the elbow and the ulna (10). As mentioned previously, Gorham’s disease mainly affects the shoulder and pelvic girdles, however it has been reported in almost the entire skeleton (1-10). One of the reasons for the delay in the diagnosis of Gorham’s disease in our patient may have been its rather unusual location i.e. the ulna. The disease can spread from one location to another, as was the case in our patient, and its spread is not limited by intervening joints. Gorham’s disease is usually not the primary diagnosis; the diagnosis is made after other more common causes of osteolysis such as metabolic, infectious, endocrine, immunologic and malignant diseases have been excluded (7, 8, 15). The pathophysiology of this disease remains unknown, although various explanations for Gorham’s disease have been suggested. Basically, bone is substituted with an expanding mass of proliferative vascular tissue resembling hemangioma or lymphoma (9). Gorham and Stout believed that the increase in local blood flow and the local pH stimulated bone resorption. They concluded that trauma could induce the formation of vascular granulation tissue and start hemangiogenesis. They believed the increase in the number of osteoclasts was not necessarily the cause of the disease (5, 8-16). Devlin et al. examined bone marrow from patients with Gorham’s and found an increased number of multinucleated cells resembling osteoclasts. They concluded that the increased osteoclastic activity causes the disease (17). In addition, thyroid T-cell activity, calcitonin (17), elevated levels of serum IL-6 up to 7 times (18), enzymatic activity of perivascular mononuclear cells (19) and elevated Macrophage Colony Stimulating Factor and Receptor Activated Nuclear Factor KB Ligand (8) have all been shown to play a role in the pathogenesis of the disease. According to previous reports, the disease is seen in all age groups and in both men and women, however it seems more common in patients under 40 years of age. Gorham’s disease is not genetically transmitted. In case of involvement of the ribs, scapula and the vertebrae, it can spread directly to pleural lymphatic vessels and result in chylothorax (20). Without surgical treatment in this case the mortality and morbidity can be very high (1). The disease is manifested by gradually increasing pain which results in anatomic changes that cause local symptoms. Sometimes the disease is discovered after a pathological fracture (1). There is no specific treatment for the disease and currently treatment is aimed at preventing osteoclastic activity (10).Current methods of treatment includes radiotherapy and chemotherapy with alpha-interferon (4, 7, 21, 22) which is not applicable in all cases (10). These modes of treatment are effective in the proliferative process by preventing angiogenesis (8, 15, 23). According to some authors, the disease may regress spontaneously (7, 9, 15, 24) or sometimes after radiotherapy (25). Radioisotope scans may show increased uptake in the proliferative stage, however later with absorption there will be reduced uptake on the isotope scan. Sometimes the disease progresses relentlessly and becomes unresponsive to current modes of treatment (15, 22, 26).As many experts recommend palliative treatment for this syndrome, in this case we too, have opted palliative therapy for our patient (arm cast) which has proved successful. The present case shows that Gorham’s disease may affect the ulna primarily and spread to adjacent bones despite the fact that there are no such reports in the literature.
